# A Systematic Literature Review of Cultured Meat Through the Conceptual Frameworks of the Entrepreneurial Ecosystem and Global Value Chain

**DOI:** 10.3390/foods14050885

**Published:** 2025-03-05

**Authors:** Chiara Benussi, Antonella Samoggia

**Affiliations:** Department of Agricultural and Food Sciences, University of Bologna, Viale Fanin 50, 40125 Bologna, Italy; antonella.samoggia@unibo.it

**Keywords:** cellular agriculture, alternative protein, policy, management, cultivated meat, food technology, cell-based meat

## Abstract

Cultured meat (CM) is currently experiencing a surge in popularity, primarily due to its promise to produce animal-based products with a lower environmental impact and a higher level of animal welfare. Although CM production remains limited and lacks pre-market approval (except for Singapore and the USA), recent technological advancements have been notable. A greater number of stakeholders, including biotechnology companies, start-ups, private investors, NGOs and big agrifood companies, are entering the CM value chain. This paper aims to aggregate, synthesize, and analyze existing studies on the CM value chain to highlight the characteristics, methodologies, and topics they address. Our secondary purpose is to analyze elements emerging in terms of global value chain dynamics. To do so, this study applies a conceptual framework based on the interplay of the Entrepreneurial Ecosystem and global value chain frameworks. This systematic literature review identifies 43 studies and shows that the most addressed topics are regulations on pre-market approval and labelling, technological progress, the use of patents, the availability and sources of funding, and actors’ roles in the CM market. The analysis and discussion of these findings highlight key aspects of the CM global value chain and present further areas of research to investigate the governance of the chain.

## 1. Introduction

Animal-based products have faced criticism due to the negative externalities associated with their production and consumption [1]. Meat, in particular, has been questioned for its unethical practices, especially concerning animal welfare, potential health risks, and environmental impacts, including land use and greenhouse gas emissions [2]. Researchers and businesses are working on the development of alternative sources of protein to meet the rising demand for sustainable and healthy food [3,4]. Among alternative proteins, cultured meat (CM) offers a potentially more sustainable alternative to conventional meat [5].

Cultured meat, also known as in vitro, lab-grown, cultivated, or cell-based meat, involves the production of animal cells through tissue engineering and biotechnologies instead of using livestock [6]. The last few years have seen an outbreak of academic, entrepreneurial, and political interest in this novel food technology thanks to its promises of delivering cruelty-free meat while using less land and emitting lower CO_2_ compared to traditional meat products [7,8,9,10]. The data indicate a growing number of companies working on cultured meat and seafood and investments in the sector by both private and public entities, which has reached a peak in 2021 with USD 5.6 billion invested [11,12].

The topic of CM innovation has gained increasing attention in the literature, especially concerning technology, food safety, and consumer acceptance. Past studies have examined the CM value chain too, mainly focusing on its structure [13], stakeholder involvement [14], and market dynamics [15,16]. However, the literature lacks a systematic review of previous knowledge on the cultured meat value chain. A systematic literature review will serve to aggregate, synthesize and analyze previous knowledge to investigate the complexities of this novel food technology and present further areas of research. Thus, this study aims to review the state-of-the-art academic literature on cultured meat value chains. This is carried out by applying the Preferred Reporting Items for Systematic Reviews and Meta-Analyses (PRISMA) guidelines. Moreover, through a conceptual framework that integrates the Entrepreneurial Ecosystem (EE) and the global value chain (GVC) frameworks, this study discusses what emerges from the reviewed studies in terms of the governance and power dynamics of the CM value chain. The interplay of the EE and GVC frameworks enhances the understanding of this emerging global value chain, particularly through the examination of food chain relationships and entrepreneurship.

The current research makes a twofold contribution. First, it examines the key characteristics of the reviewed studies on the CM value chain and highlights potential areas for further investigation. Second, it offers a qualitative content analysis of the selected literature highlighting topics discussed related to the structure, governance, and power dynamics within the CM GVC. Investigating these aspects offers valuable insights into the current dynamics of the CM chain and its future development. This review is particularly relevant due to the transformative potential of CM in reshaping global food systems, as well as the novelty of the field.

## 2. Conceptual Framework

### 2.1. Entrepreneurial Ecosystem

The EE is defined as a key tool to describe the functions and the complex network of relationships between actors that support the entrepreneurial performance of an ecosystem [17]. It emerged as a framework to understand how a favourable context for entrepreneurship is built and developed [18]. The rationale behind this framework is rooted in the relevance of entrepreneurship in boosting the economy and promoting innovation and social development [17]. The metaphor “ecosystem” serves to move away from an individualistic understanding of entrepreneurship to a collective idea of entrepreneurship as a result of social, cultural, institutional, and economic forces [19]. The present paper adopts Isenberg’s Six Domains Model, a well-known and comprehensive model for EE analysis [18,20]. According to Isenberg, six domains interact to define an Entrepreneurial Ecosystem’s policy, which encompasses regulation and political context; finance, including private and public investments; human capital, referring to the skills of the workforce; market, which involves actors in the market space; support, covering infrastructure and energy; and culture, including actors shaping perception [21]. Inside each domain, there is a set of functions and networks to consider, which interact with each other.

This paper employs the EE to address the intrinsic entrepreneurial dynamics of the CM sector. Entrepreneurs have been involved in the CM chain mainly by participating in the establishment of CM start-ups. These start-ups support the system by advancing technological knowledge and promoting CM cultural acceptance. The application of the EE framework supports the vision of the entrepreneurial environment as a pivotal element of the CM chain, as pointed out in the literature [22].

### 2.2. Global Value Chain

The GVC was developed by a group of researchers led by G. Gereffi in the 1990s [23]. Their studies used the expression Global Commodity Chain to indicate a conceptual framework aiming to analyze the increasing geographical dispersion of commodity chains [24]. Researchers elaborated on this concept by adding a focus on value creation and distribution [23]. This framework was named the global value chain and served to analyze value-added activities and governance dynamics along the value chain [25,26]. The GVC has proven to be a lens of analysis for merging meso and macro aspects of the global economy in multiple sectors while providing an exhaustive map of stakeholders and production activities to understand the geographical distribution of actors, their roles along the chain, and how inter-firm relationships are built and led along the value chain [27].

One of the main dimensions of the GVC analysis is chain governance, defined as “the actions, institutions and norms that shape the conditions for inclusion, exclusion and mode of participation in a value chain, which in turn determine the terms and location of value addition, distribution and capture” [28] (p. 667). Strictly connected to governance is the element of the analysis of power distribution, which looks at the power dynamics between actors and how power contributes to shaping the GVC governance. The seminal GVC studies distinguished between two types of chain governance based on the concentration of power in the relationship between producers and distributors: producer-driven and buyer-driven value chains [24]. Later, researchers shifted to a more comprehensive understanding of GVC governance based on the analysis of inter-firm relationships as links and networks. Furthermore, power analysis was broadened by including power relationships among different actors, not only at the firm level, but less defined and more diffuse, resulting in four types of power: bargaining, institutional, demonstrative, and constitutive power [28]. Value chain governance is understood based on the complexity of transactions, the flow of knowledge and information, and the capabilities of suppliers. These three variables are determined by the technological features of the products and the settlement of standards and certification [23]. Thus, aspects such as certification, R&D, regulation, and intellectual property are often considered while discussing the governance of the GVC.

The GVC provides a comprehensive framework for the CM sector for the following reasons. First, the CM chain is identified as a globalized value chain due to its inter-firm relations with actors located worldwide. Second, the GVC acknowledges technology as one of the factors influencing GVC development, with particular emphasis on analyzing the structure of technology-intensive value chains and the impact of technology on their upscaling and governance [24]. As a highly technological chain, the CM chain aligns with this focus within the GVC framework. Finally, the analysis of the CM GVC contributes to the extensive body of GVC literature on agrifood chains, which includes early wine and coffee GVCs [29,30,31].

### 2.3. The Interplay Between the EE and GVC Frameworks

**Figure 1:**
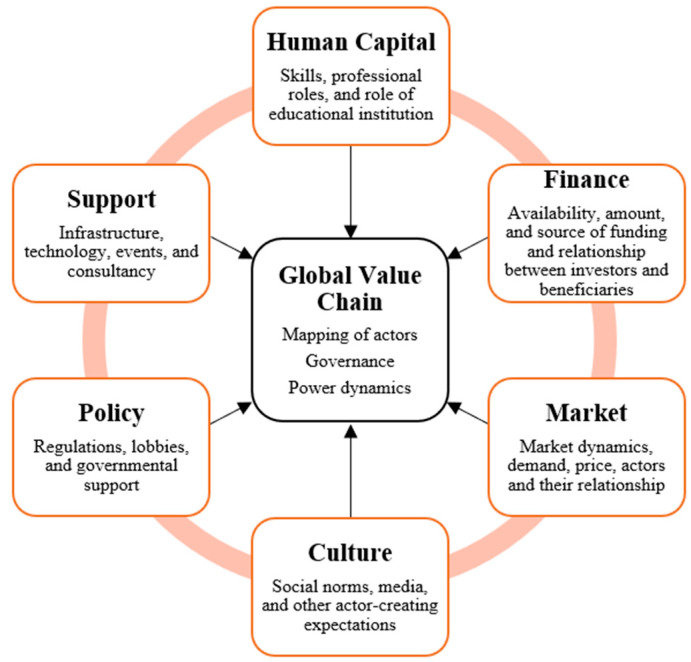
Visual representation of the conceptual framework based on the interplay between the Entrepreneurial Ecosystem and global value chain.

If applied separately, both the EE and the GVC may provide a limited perspective of the CM value chain dynamics. First, the EE framework overlooks power distribution and governance issues within the chain. Second, the novelty of the CM industry and its early stage of development limit a deep GVC analysis. Therefore, the present paper applies a combined framework that offers a nuanced understanding of the functions and actors shaping the CM value chain by leveraging insights from both the EE and GVC approaches. The adopted conceptual framework supports the analysis of internal supply chain dynamics, such as market structure, technological support, and human capital, alongside external supply chain factors, such as policy, culture, and finance. Each domain of the EE enhances our understanding of the CM value chain by addressing crucial elements of the GVC framework (Figure 1). The market domain focuses on how the CM market is structured, focusing on suppliers, producers, and distributors and its future growth. This analysis supports the GVC framework by providing insights into actors’ mapping and power dynamics along the chain. The finance domain analyzes the availability, sources, and scale of investments that may be critical for CM companies to scale up and advance in their R&D efforts. In GVC terms, this highlights the power relationships between CM companies and investors. The support domain investigates the role of technological development and infrastructure in the CM value chain. As technology is a key element to understanding GVC, it is essential to explore these dynamics. The human capital domain examines the professional roles and skills available in the CM chain, emphasizing how the availability of a skilled workforce may influence GVC upgrading and so affect GVC governance structures. The policy domain studies the regulatory frameworks, the political dynamics, and the influence of interest groups and institutional actors in supporting or hindering the progress of the CM industry. This domain impacts GVC participation and offers insights into the distribution of power across the chain. Finally, the culture domain addresses the social acceptance of CM, the role of the media, and how cultural functions can either facilitate or hinder the public acceptance and support of CM products. These cultural elements shape value creation and governance within the CM value chain [32].

## 3. Methodology

### 3.1. Data Collection

**Figure 2:**
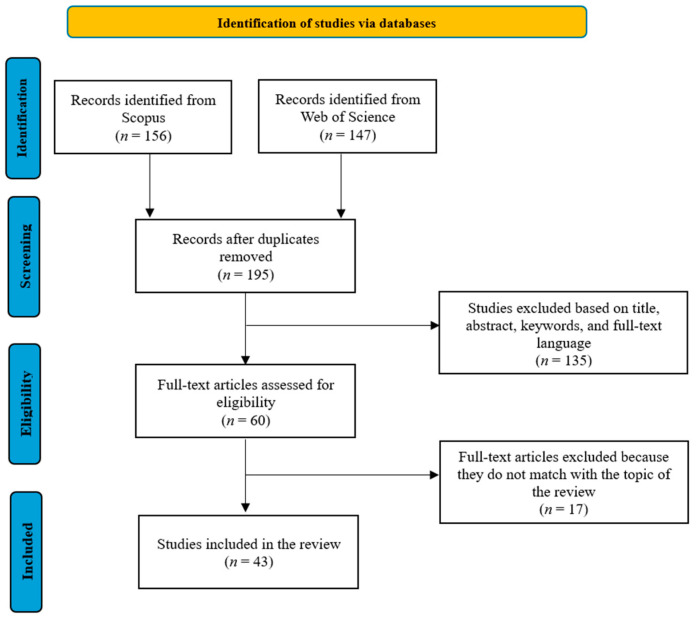
Collection and selection of studies according to the Preferred Reporting Items for Systematic Reviews and Meta-Analyses (PRISMA) flow diagram.

The present study systematically reviews the academic literature by applying the PRISMA guidelines, with the search strategy process illustrated in Figure 2 [33].

The data collection was conducted using two search engines (Scopus and Web of Science), known for their comprehensiveness of peer-reviewed articles. A combination of words was used as search strings in each of the two databases (Table 1). The search string included three lists of terms connected by “AND”. The first list was as comprehensive as possible by including all the expressions used to indicate products from cellular agriculture (such as cultured, in vitro, cultivated, cell-based, and lab-grown products) in combination with the types of animal protein (meat, seafood, and dairy). The second list covered all terms related to the topic of policymaking, regulation, business models, and the development, governance, and structure of the value chain. Finally, the third list included two terms to limit the research to studies only applied to the food and feed system.

The following steps involved the evaluation of studies. Studies were excluded if they were duplicates if the full text was not available in English or Italian, and if they focused on consumers; as such, these studies fell outside the focus of this research. Articles, reviews, and chapters were included. The articles were screened based on their title, keywords, and abstracts. The resulting 60 studies went through a detailed full-text reading, and 17 studies were omitted as their content did not align with the aim of the research. Finally, 43 studies were included in the final body of the literature analyzed. Data selection was completed between December 2023 and January 2024. The full list of studies selected in the present literature review is available as Supplementary Material.

### 3.2. Data Analysis

The analysis of the selected articles included two parts. The first part focused on the analysis of the studies’ characteristics, and relevant data were extracted based on the year of publication, journal of publication, the type of product addressed, the type of data presented, and the methodology used to collect data. Detailed information on the characteristics of the studies is available in Appendix B.

The second part of the data analysis combined a qualitative and quantitative content analysis based on the conceptual framework. NVivo 12 software was used to systematically manage the data. The analysis applied a codebook developed by the authors (Appendix A), which provides a structured approach to coding. The codes and sub-codes were identified through a multi-step process. First, the codes were derived from the conceptual framework, with each domain of the EE framework assigned a corresponding code. Two additional codes—actors and geographical location—were added to capture the key actors involved and the geographical scale of the CM chain. Second, the definition of sub-codes was based on the analytical interpretation of the EE domains using the GVC framework as a lens. A review of previous studies on EE informed the identification of sub-codes deemed as key elements for understanding each EE domain [17,18,20,32]. Finally, the list of sub-codes was consolidated with the addition of relevant sub-codes that emerged from a word frequency query run in NVivo12. The coding strategy and the coding scheme were discussed by the authors through periodical meetings to avoid ambiguities and enhance coding reliability. One researcher conducted a preliminary coding of the studies to ensure the uniform interpretation of the data, which they then consolidated with a second author to solve any outstanding issues and reach a consensus.

With the coding results, a comprehensive content analysis was conducted, incorporating both quantitative and qualitative approaches. The quantitative analysis allowed the authors to identify the frequency and distribution of key themes within the reviewed literature. The qualitative analysis allowed the authors to interpret the relevant topics emerging from the reviewed studies in alignment with the objectives of the present research [34].

## 4. Results

### 4.1. Main Themes and Geographical Focus

**Figure 3:**
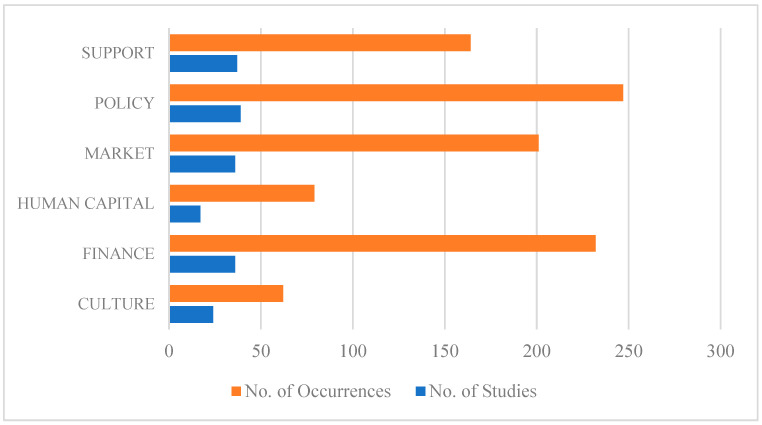
Number of studies with at least one occurrence of the selected domain and number of occurrences per domain.

The results show that there are four EE domains that mainly occur in the reviewed literature support, policy, market, and finance, with an average of 37 articles discussing these themes (Figure 3). In contrast, a smaller number of articles mention human capital and culture, suggesting that these topics are not addressed in the reviewed literature. Detailed information on the distribution of sub-codes and the temporal distribution of codes is discussed in Appendix C.

**Figure 4:**
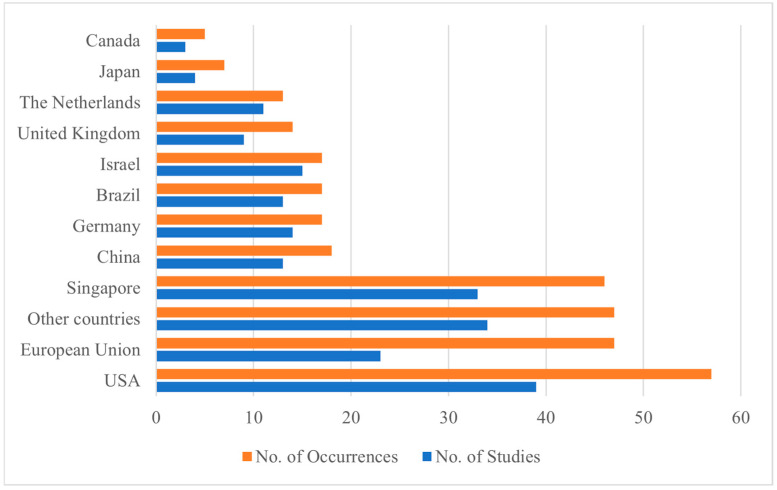
Number of articles with at least one occurrence of the selected country and number of occurrences per country.

The result highlights that the CM chain is mostly discussed in a small group of countries, namely highly technological and high-income countries, as only 15% of occurrences mention other countries (Figure 4). The USA emerges as a key player in the CM sector as the most mentioned country. The European Union is often cited, especially in terms of regulation and politics, since more than 70% of EU occurrences fall into the domain of this policy (Appendix C). Among the studies analyzed, certain research adopted a general perspective on the European Union, whereas others delved into only one European country, such as Germany and the Netherlands. Going beyond Western countries, Singapore is the most referenced Asian country: this is clearly due to its pioneering role in authorizing CM commercialization and consumption and its pivotal entrepreneurial environment.

### 4.2. Market

Most authors classify the CM market as in an infant state due to the absence of large-scale production facilities and the limited sale of CM products. The commercialization of CM is hindered by the requirement to obtain pre-market authorization from food safety authorities, as CM is categorized as a novel food (see Section 4.6.1). Scholars also highlight the different stages of development between the CM market and the plant-based analogue market, with plant-based products taking the lead thanks to the lower technological and social changes needed compared to CM [35]. Furthermore, the reviewed studies have different perspectives on the potential market penetration of CM products. While some authors have limited expectations of the up-taking of CM products over conventional products and expect CM to be a premium product [36,37], others forecast a greater role for CM in protein transition [38,39]. Nonetheless, new business opportunities may arise with the further development of the market [15,40].

#### Structure of the Market

The various stages in the CM value chain have been defined as the following: first, the value chain involves suppliers of raw materials and technologies; second, it includes CM producers and processors; and finally, it revolves around the distribution, commercialization, and consumption of the products [13]. Suppliers are upstream of the CM GVC by providing raw materials, such as culture media, embryonic cells, and cell lines, as well as supplying equipment, like bioreactors. Suppliers of raw materials are mostly B2B biotechnology companies and are based in technologically advanced countries. As such, CM processors emerge as key actors in the value chain. According to one study [41], about 60 CM companies are working on a wide range of products, which vary from the most popular foods like hamburgers to seafood and foie gras. CM processors are mostly small start-ups working on a business-to-consumer model backed up by private investors [14,42]. The literature also mentions that while numerous small companies focus on technology development and raw material supply, some CM processors are vertically integrating chain activities from production to marketing [40,43].

The literature dedicates hardly any attention to the distribution and commercialization stage of the value chain. This is probably due to the limited number of commercialized products and limited number of distribution channels, as only a few Singaporean and USA restaurants and supermarkets are commercializing the few market-approved CM products [44]. Yet, some authors foresee that this stage of the value chain could be strengthened by closer collaboration between CM and conventional meat companies [43]. In fact, the established relationship between conventional meat companies and retailers and food services may help CM companies enter the retail space.

Finally, the literature largely points out how consumer reluctance plays a crucial role in the uncertainties linked to the uptake of CM products in the food market [16,45]. The authors mention issues such as low taste and poor sensory characteristics as the pressing force on consumers [45,46,47]. Price remains a critical aspect to consider when discussing the future market penetration of CM products; while huge steps on price reduction have been reached by CM companies, they are far from being competitive with conventional products [35,47,48].

### 4.3. Finance

Past studies recognize the pivotal role of funding in advancing R&D and start-ups, recognizing investors as key actors for chain development [14,44]. The large volume of investments required for R&D and start-up establishments highlights the relevance of financial resource availability for investments in CM GVC development. Only in the period between 2019 and 2020 did the capital invested in the cultured meat sector reach more than USD 350 million [41].

#### Public Versus Private

The literature identifies private investors as the main source of investment for CM processors [49]. A recent study on investor perceptions of CM revealed their enthusiastic aptitude towards the potential upscaling of cellular agriculture, seeing many financial opportunities [14]. Among private investors, venture capital is the main source of funding for CM start-ups [40,44,49,50,51,52]. While most of the studies mention who the main ventures involved are and the amount of capital invested, only a few studies delve deeper into the rationale behind private investments in CM and the outputs. For example, one study stresses that the flow of private capital in CM start-ups has allowed the status of cellular agriculture to be upgraded, boosting trust in its further development [49]. Some authors associate the involvement of private investors in CM companies with what they delineate as an “investment culture of disruptive innovation” [53]. CM comes as a “disruptive innovation” for its potential to displace established businesses, namely conventional meat producers, by either rendering their production methods obsolete or compelling adaptation to the emerging market competitor. The authors observe a parallel between the engagement of private investors in the CM sector and other venture-backed disruptive business models like Spotify, Uber, and plant-based companies.

The literature points out the increasing participation of big agrifood companies in financing the CM chain. Many authors mention big agrifood companies, such as Cargill, and large conventional meat processors, such as JBS and Tyson Foods, as investors [40,51,54,55]. The engagement of large conventional meat producers in financing the CM sector dates back to 2017–2018, showing an established relationship between the conventional meat and CM sectors [44,49].

Parallel to venture capital funding and large agribusinesses, angel investors and philanthropists have joined the race towards CM technology development, mirroring the trend observed within the plant-based sector. Studies mention the participation of famous entrepreneurs and individual investors, such as Bill Gates, Richard Branson, PayPal billionaire Peter Thiel, and Google co-founder Sergey Brin [44,56,57].

The literature mentions that a few national governments have joined the race to fund CM research [49]. Notably, the Dutch government and the American public agency NASA were the first public bodies to finance research in cellular agriculture [57,58,59]. However, the participation of governments is still limited: only a small group of countries finance CM research with limited resources compared to private investments. Indeed, a part of the reviewed literature emphasizes the lack of public participation as a critical point for the development of a fair and open CM value chain and welcomes a greater role of public institutions in financing the development of the CM sector [35,36,60]. For example, some researchers [61] stress the importance of public investments to ensure long-term research endeavours in contrast to the prevailing model of private investment based on rapid and large-scale returns, which carries along risks of instability over time and may impede access to technological advancements.

### 4.4. Support

The reviewed literature discusses the support domain mainly in terms of technology, partially addressing other functions, such as energy use in CM production. The analyzed literature lacks agreement on the energy impact of CM production, suggesting that it depends on technological advancements, the upscaling of production, and the type of energy source used [37,61,62,63,64].

#### 4.4.1. The State of Technological Progress in Cellular Agriculture

Even if studies on food technology are out of the scope of this study, the state of CM technology often emerges as a relevant determinant for the future of these products and the transition of the CM sector from a technological niche to a market niche [35,41]. The literature mentions the need for CM technological advancements to reduce cost production, improve sensory properties, and replace the use of fetal bovine serum. Another upcoming technological challenge is the upscaling of production, as it will serve to determine if CM can be produced at a lower cost and with higher volumes [15,47,55,65].

#### 4.4.2. Ownership of Technological Innovation

A key topic addressed by past literature is the ownership of technological innovations in the CM sector, where patents are highly used [35]. In a previous study, 78 patent families were counted for CM-related technologies published between 1999 and 2022 [44]. This study highlights the high use of patents by the private sector compared to the limited use by universities and public research bodies. Other authors point out that while interviewees welcome greater participation in CM innovation, most of the industry-affiliated interviewees expect that the protection of intellectual property will remain a priority in the CM sector [49]. CM start-ups use patents to protect their competitive advantage and to guarantee a return to private investors [49,50]. Thus, the engagement of private investors, especially venture capital, directly influences the choice of CM start-ups to use patents [51]. Opinions differ on the outcomes of the high use of patents in the CM chain; while some authors argue that competitiveness among CM start-ups, increased by closed access to intellectual property, may allow companies to attract top professionals and stimulate innovation [61], others argue that closed access to innovation may also limit participation in the GVC and hinder the overall development of the chain [35]. However, opening access to CM technologies would not necessarily increase participation in the CM chain, as it also depends on other factors such as training and education [61].

### 4.5. Human Capital

#### 4.5.1. Skills and Professional Roles in the Cultured Meat Value Chain

An increasing number of professionals have entered the CM value chain, persuaded by the increasing job opportunities and its moral disposition [40,56]. As the CM industry develops, the reviewed literature expects that the CM GVC will create new job opportunities, especially in high-income countries [40,66]. Skills in disciplines such as biology, food engineering, and chemistry are fundamental in the production stages of the chain [46,54]. At the same time, a few studies mention the increasing job opportunities for professionals in marketing, supply chain management, and business strategy [46,54]. The set of business-oriented capabilities may play a pivotal role in the development of CM start-ups by bringing added value in the downstream stage of the value chain [46]. However, the literature discusses how the demand for highly developed technological skills may prevent participation in the value chain if not combined with adequate educational or training programmes [54]. Thus, the novelty and complexity of CM technologies may require educational institutions to implement their offer with specific CM educational programmes [54]. These results confirm that the CM chain requires highly specialized professionals, especially for CM production, and the investment in human capital will determine the CM chain’s further development.

#### 4.5.2. Educational and Training Programmes for Farmers

Diverse opinions emerge from the literature on the future of livestock producers [45]. The literature discusses how the further development of the CM chain may impact farmers and which roles they could play inside the chain. On the one hand, a few authors mention that the potential upscaling of CM may decrease the price of CM products, making them more cost-competitive to conventional meat, thus threatening farmers’ livelihoods, especially small-scale farmers who are already made vulnerable to market dynamics [36,63,67]. On the other hand, some studies welcome the participation of farmers in the new value chain as suppliers of raw materials, even if it may be costly and may require farmers to acquire new skills [55]. Thus, the possibility of farmers entering the CM chain as suppliers will depend on the upscaling of the CM GVC and farmers’ capabilities. Some studies call for training opportunities or specific educational programmes to support farmers participating in the new value chain or to transition to different activities [40,54,55]. Some authors highlight the need for policy interventions to address and support farmers in the transition to cellular agriculture [52,68].

### 4.6. Policy

#### 4.6.1. Regulations

The regulations applied to CM products concern a few application scopes. First, most of the regulatory discussion concerns the pre-market assessment regulation [60]. CM food safety is a major concern, and regulations address it by classifying CM as a novel food and, consequently, imposing pre-market assessments. Overall, the reviewed literature discusses food safety regulations for a limited group of countries, including the USA, Singapore, and the European Union, as they are the most involved in the CM sector so far. Second, several studies delve into the matter of labelling CM products, which is a challenge shared with plant-based alternatives [60]. The debate around meat-sounding labels is still ongoing, and it involves the possibility, or not, of calling products from cellular agriculture with meat-sounding names. Lastly, the reviewed literature reflects on the ontological definition of meat and the nature of the product, noticing that most of the definitions of meat, as defined by the European Parliament and by the American Meat Science Association, would not apply to CM [47,69]. Furthermore, there is inconsistency among researchers and companies on what to call it (cultured, cell-based, in vitro, etc.), and it varies according to marketing strategies and consumers’ perceptions [39,53,67,70]. The past literature acknowledges that the controversy around labels and meat definition may slow down the development of the CM value chain, prevent its commercialization, and mislead consumers [60,70,71].

#### 4.6.2. Politics

A diverse range of actors are engaged in the political context of the CM GVC, shaping CM-related policies and driving developmental objectives.

First, studies mention regulatory bodies and national agencies as the policymaking bodies responsible for CM regulation. Past studies highlight that the decision over which regulatory bodies should deal with regulations affecting CM is one of the challenges behind CM regulation [52]. So far, most of the governmental bodies addressing the CM pre-market assessment are food safety agencies, such as the EFSA at the EU level. In the US, after an extensive debate, the Food and Drug Administration (FDA) and the US Department of Agriculture (USDA) found an agreement to jointly collaborate in the supervision of CM food safety, production and commercialization [16,59,60]. According to the literature, the question of who should regulate CM is linked to both the novelty of the topic and the challenge of regulating biotechnologies per se [72].

Second, the literature mentions national governments and supranational organizations, such as the EU, as key stakeholders in the CM GVC due to their financial support to CM research (see Section 4.3) and their political endeavour to address CM questions, such as policies. A few studies lament the limited governmental support to the CM sector so far, except for the Singapore government; strong governmental support has facilitated the participation of Singaporean companies in the CM GVC through the provision of investments, the development of a nourished professional community, and the expansion of the market [43].

Third, past studies highlight the pivotal role of philanthropic associations and NGOs in advocating and stimulating the development of the CM sector by organizing conferences, providing funds, and agglomerating scientific and technological knowledge [35,49]. Studies specifically mention organizations active in alternative protein promotion, such as New Harvest and the Good Food Institute, as well as environmental and animal rights associations, such as People for the Ethical Treatment of Animals (PETA) [37,65].

Finally, lobbies of farmers and agricultural associations emerge as a political force in the CM regulation debate. They enter the political debate on labelling by strongly positioning against any meat-sounding label regulation [70]. According to some authors, the pressure of farmers’ associations may limit governmental engagement in the CM sector [60].

### 4.7. Culture

The past literature highlights relevant insights into the cultural aspects of CM. Media engagement in creating expectations and perceptions around CM may play a crucial role, alongside alternative proteins in general [43,73]. The literature acknowledges that the media has covered the topic since the first public event in 2013, which was addressed by the media with a positive attitude, especially in Anglo-Saxon countries [48,57,66,74]. Overall, the reviewed literature claims that media coverage has both supportive and critical opinions [41,48]. Moreover, one study points out a lack of controversial voices in the media coverage [63]. In particular, they see positive media coverage of news coming from industry-affiliated scientists and CM company representatives, the ones mostly benefiting from a positive public perception of CM [63]. Nonetheless, it should be noted that this publication dates back a few years ago and may not respect current feelings towards CM. Other authors emphasize the significance of policymakers in addressing the communication and promotion of CM and ensuring the transparent dissemination of science-based information to the public [60].

Table 2 presents a schematic representation of the main results emerging from the content analysis of the reviewed studies distinguishing between the six EE domains.

## 5. Discussion

The present study presents a comprehensive review of the existing literature on the CM value chain and aims to explore the structure of the CM GVC, its governance, and its power dynamics as emerging from the reviewed studies.

### 5.1. Actors in the GVC

Thanks to the EE and its ecosystem approach, the present study reveals the multi-actor structure of the CM GVC. While a previous study [13] represents the CM GVC mainly focusing on the actors involved in the production and distribution stages, the present results assert that other relevant stakeholders engage within the CM ecosystem, each of them with a distinct role in the chain. A few considerations may be taken based on the multi-actor structure of the CM GVC. First, CM companies are distinguished as the most active players in the value chain as the main beneficiaries of funding and promoters of innovation and technological development. Second, the literature lacks an investigation of the upstream stage, especially the role of suppliers and their relationship with CM companies. Third, middle food system actors, such as retailers and HORECA, are overlooked by the literature. While this is strictly related to the lack of commercialization of these products, it lacks a deeper conceptualization of the role of these actors in bringing CM to consumers. This indicates that the engagement of retailers in the chain value of alternative proteins is closely tied to products that tend to be market-ready, and thus, pre-market approval is a must for the active participation of retailers in the CM GVC. However, delaying the establishment of connections with retailers may reduce the potential for CM companies to successfully penetrate the market. Fourth, the present research highlights the role of stakeholders, such as private investors, national governments, and NGOs, in influencing the development of the CM GVC. They affect the EE around CM in the policy, finance, culture, and support domains. Finally, the results show that farmers’ positions inside the GVC remain uncertain. A deeper discussion on the interaction between CM GVC and the agricultural system may be implemented to create a cooperative environment among farmers and CM companies.

### 5.2. Governance

Governance depends on the complexity of transactions, the flow of knowledge and information, and the capabilities of suppliers. However, the reviewed literature shows a lack of discussion on these topics due to CM’s novelty. Nonetheless, the results provide other elements that are able to shed light on the CM GVC governance configuration, and the authors suggest policies, human capital, and support as key EE domains shaping the chain governance. First, regulatory frameworks are a key determinant for the production and commercialization of cellular agriculture products. Currently, policy scenarios around CM, as well as alternative proteins, are still undefined, and questions surrounding the food safety and labelling of these products are still under discussion, with forces such as cellular agriculture NGOs and farmers’ associations lobbying for opposing outcomes [60,71,72,75]. Furthermore, recent news has mentioned a national ban on CM commercialization in states like Florida, Alabama, and Italy [76,77]. These political actions may also influence the cultural environment around CM, increasing the polarization of opinions on this technology and hindering innovation. Second, it is essential to recognize that the marketability of CM products hinges on consumers’ acceptance, which is closely tied to technological progress that decreases production costs [16,56,68,78]. Finally, national governments have the potential to influence the development of the CM GVC. The role of public institutions may establish more balanced relationships among CM stakeholders by reducing the bargaining power of investors in CM start-ups by providing diversity in funding and promoting open access technology to foster innovation and participation [60]. National governments may support entrepreneurial activities and promote legislation that facilitates CM production and commercialization [43].

### 5.3. Power Dynamics

In the case of CM, the revised literature unfolds power dynamics, addressing different aspects. The first concern is the geographical localization of CM companies. CM companies and their suppliers are spread worldwide with a predominant concentration in a limited group of countries, such as the USA, EU, Canada, Singapore, and Israel, known for their engagement in the sector almost since its beginning and favoured by the presence of highly specialized workers and significant investments in food technologies [39,41,43,50]. Furthermore, regulatory differences in CM commercialization across nations further shape the entrepreneurial, human capital, and investment landscape around CM, concentrating CM development and distribution in regions with more favourable policies. Such discrepancies may also encourage the migration of expertise to countries where CM is approved. The concentration of value-added activities in high-income countries may exacerbate the disparities between high-income and low-middle-income countries by excluding low-middle-income countries from benefiting from cellular agriculture transition [58]. Consequently, some scholars welcome the implementation of international policies designed to protect low-middle-income countries from the adverse effects of CM chain development [40], while others argue that the advantageous position of high-income countries could positively influence the participation of low- and middle-income nations in the CM sector by enhancing workforce skills [46,52].

A second concern pertains to the involvement of incumbent actors, like big agrifood companies and venture capitals in the CM GVC through investments and partnerships [53]. Recent statistics on investments in the CM sector confirm the increasing amount of funds from large companies, such as Tyson, Danone, Nestlé and Cargill [12]. A similar pattern is seen in the plant-based market, with conventional meat brands producing plant-based products [79]. According to a few studies, the CM GVC is already showing a market-driven approach and hierarchical structure similar to the present food system and its unbalanced power dynamics [35,49,63,80]. This concentration of power may increase the bargaining leverage of private investors and multinational agrifood companies over CM processors [40,52]. This power distribution stems from the need for investments in CM start-ups to cover costs and develop technologies, and the only possibility to find them is from private investors due to the scarcity of public investments. The literature mentions that the unbalanced relationship between the two also results in closed access to technology to protect investors’ interests. However, some authors dispute this perspective, arguing that big agrifood companies may support CM start-ups in scaling up production and positioning CM in larger markets [14].

The present review supports the notion that these two aspects combine to outline a potentially unbalanced CM GVC, limiting the participation of new companies and new countries due to the power concentrations in high-income countries by big agrifood companies and venture capitalists. The EE analysis suggests that greater investment from the public sectors, open access to innovation, and lower regulatory obstacles may contribute to creating a more balanced power relationship in the CM GVC. Nonetheless, it is important to keep in mind the fact that the configuration of the CM GVC is still in progress, and future developments in investment trends, regulation, and technology development may change the governance and the power distribution of the chain [40].

### 5.4. Further Areas of Research

Promoted as an ethical and sustainable alternative to conventional meat, businesses and researchers have worked to expand the knowledge and market opportunities for CM. Now, CM is a globalized value chain, and thanks to the combined conceptual framework of the EE and GVC, this literature review captures key governance aspects of the CM GVC.

Future research may explore the following topics. First, future studies could investigate the factors influencing the participation of companies in the CM value chain, the competition and cooperation between them, and their level of vertical integration. These investigations could contribute to a better understanding of the positioning of CM companies in the GVC and the elements shaping chain governance. Second, future research may investigate the role of suppliers and retailers in CM GVC governance. Third, future investigations may focus on the business models adopted in the CM value chain. Understanding these models can provide insights into how companies generate revenue, manage costs, and scale their operations. Finally, future research may investigate the involvement of big agrifood companies and venture capital in the CM GVC. Researchers could explore to what extent their participation supports the development of CM. Altogether, these studies would offer critical insights into the power dynamics within the CM GVC.

### 5.5. Limitations

This study has two main limitations. First, it includes studies only in English. This restricts the research by excluding articles published in other languages and limits the geographical areas covered. Second, the research relies solely on two databases: Scopus and Web of Science. While these two databases are widely renowned for their comprehensiveness, expanding the sources used could add more sources of information and perspectives.

## 6. Conclusions

The present article aims to systematically review the economic and policy literature on cultured meat, seafood, and dairy through content analysis applying the EE and GVC frameworks. This study presents a rich academic body around the topic of the CM GVC, which peaked in the period between 2021 and 2023. This review is in line with previous studies, which demonstrate a growing body of the literature addressing technical, social, economic, and regulatory challenges for CM [45,64]. Overall, the CM GVC proves to be a complex and multi-actor chain, and its configuration is yet to be fully shaped. Despite the increasing participation of actors in the CM GVC, significant governance issues have been defined. The results suggest that the further development of regulations, technological progress, consumers’ acceptance, and the engagement of national governments may influence CM GVC governance. This study fits into the growing literature on CM and provides insights for policymakers, stakeholders, and researchers. Policymakers and stakeholders can use this information to understand the dynamics and factors influencing the development of the CM GVC. Researchers can use this review to explore the key issues addressed in the past literature and cover the emerging research gaps. Given the potentialities of the CM technology to shape the agrifood system, it is crucial to continue conducting studies on the CM sector, especially on the relationship between actors. Understanding the dynamics in the CM GVC is essential for supporting a fair, open, and sustainable development of the CM GVC.

## Figures and Tables

**Table 1:** Description of the search string and studies identified in the literature databases.

Search String	Database	*n* of Studies Identified
(TITLE-ABS-KEY (“cellular agriculture” OR “cultured meat*” OR “in vitro meat*” OR “synthetic meat*” OR “lab grown meat*” OR “cultured meat-based protein*” OR “cultivated meat*” OR “cell-based meat*” OR “cultured seafood*” OR “in vitro seafood*” OR “synthetic seafood*” OR “lab grown seafood*” OR “cultured seafood-based protein*” OR “cultivated seafood*” OR “cell-based seafood*” OR “cultured cheese” OR “in vitro cheese” OR “synthetic cheese” OR “lab grown cheese” OR “cultured cheese-based protein*” OR “cultivated cheese” OR “cell-based cheese” OR “cultured dair*” OR “in vitro dair*” OR “synthetic dair*” OR “lab grown dair*” OR “cultured dairy-based protein*” OR “cultivated dair*” OR “cell-based dair*”) AND TITLE-ABS-KEY (“food system transformation” OR “food system innovation” OR “food system transition” OR “sustainable food system*” OR “food chain transformation” OR “food chain innovation” OR “food chain transition” OR “sustainable food chain*” OR polic* OR politic* OR legislation OR law* OR “regulatory framework*” OR “regulatory action*” OR “regulatory approach*” OR “government regulation*” OR “value chain*” OR “supply chain*” OR “agri-food chain*” OR “agro-food chain*” OR stakeholder* OR governance OR “chain manage*” OR “business model*” OR “ business development*” OR “incumbent firm*”) AND TITLE-ABS-KEY (food* OR feed*)	Scopus	156
	Web of Science	147

**Table 2:** Schematic representation of the main results based on the content analysis of the EE domains.

Market	Finance	Support
- Infant state of the market - Pivotal role of CM start-ups in promoting technological progress - Lack of analysis of the role of retailers and distributors in the value chain - Market penetration uncertainty - Lack of consumer acceptance and pricing Issues	- Crucial nature of funding for R&D in CM technology - Investment culture of disruptive innovation - Private funding from venture capitals, angels, and philanthropists - Involvement of big agrifood companies in the CM value chain as investors - Limited public funding	- Lack of agreement on the energy impact - Technological progress as a key determinant of the future of CM - Key technological challenges in cost reduction and upscaling - High use of patents in CM innovations
**Human Capital**	**Policy**	**Culture**
- Request for highly specialized professionals - Potential to increase the number of employees in the sector - Uncertainty around the role of farmers in the value chain	- Regulations for CM focus on pre-market assessment, food safety, and labelling - Limited government participation in addressing regulatory issues and supporting business development - Political context influenced by farmers’ lobbies and NGOs	- Supportive and critical media coverage - Need to ensure the transparent dissemination of science-based information

**Figure A1:**
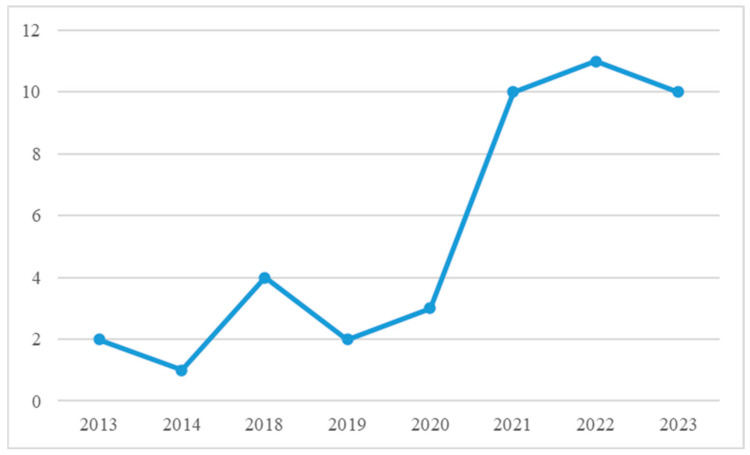
Temporal distribution of the reviewed studies.

**Figure A2:**
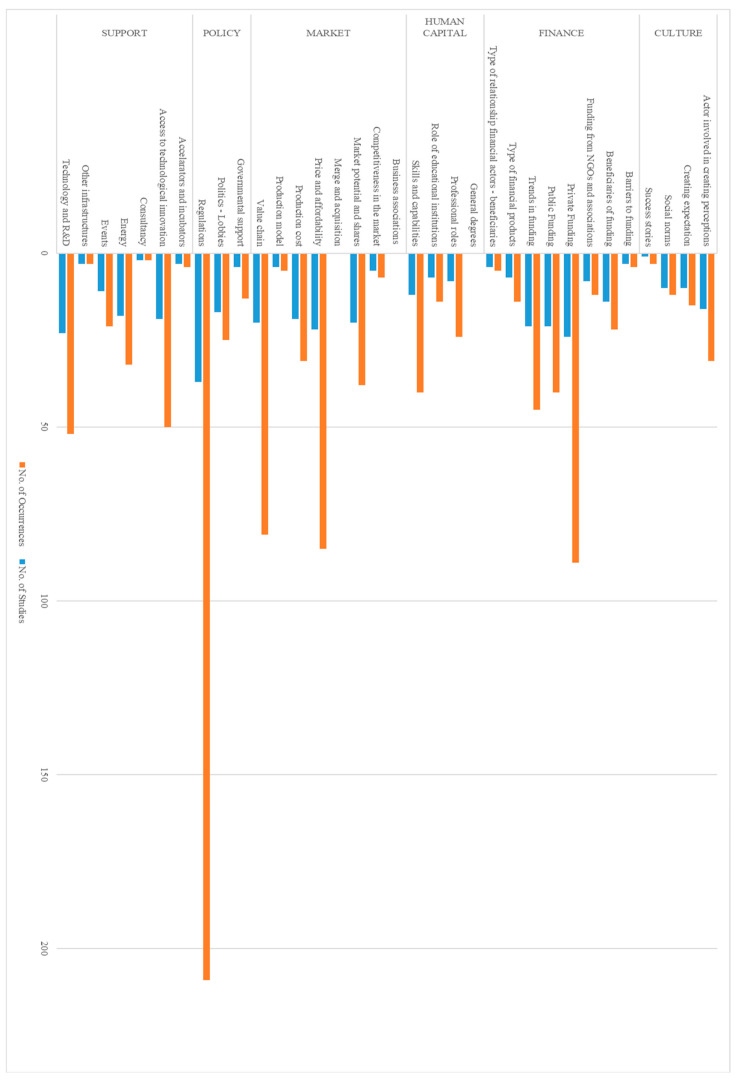
Number of articles with at least one occurrence of the selected codes and sub-codes and the number occurrences per code and sub-code.

**Figure A3:**
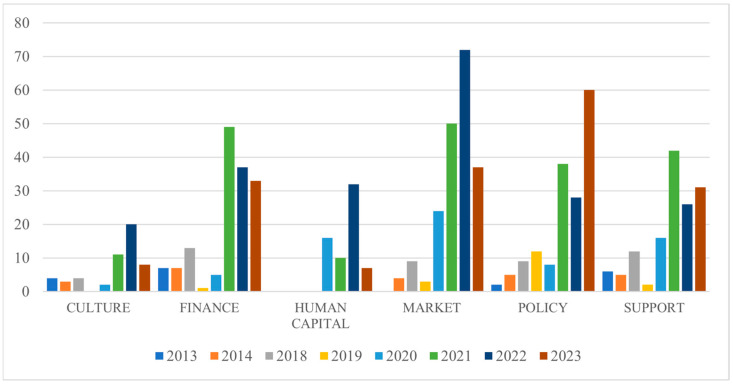
Distribution of occurrences by year of the publication of the study.

**Figure A4:**
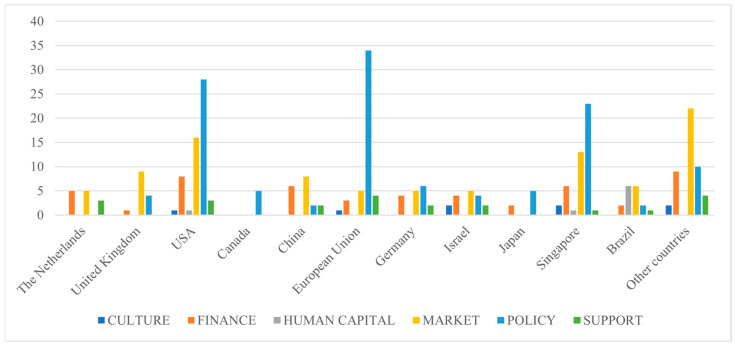
Number of co-occurrences per country and domain.

## Data Availability

No new data were created or analyzed in this study. Data sharing is not applicable to this article.

## Supplementary Materials

The following supporting information can be downloaded at https://www.mdpi.com/article/10.3390/foods14050885/s1. Table S1. List of reviewed studies included in the literature review.

## Abbreviations

The following abbreviations are used in this manuscript:

CM	Cultured meat
EE	Entrepreneurial Ecosystem
GVC	Global value chain
PRISMA	Preferred Reporting Items for Systematic Reviews and Meta-Analyses

## Appendix A

**Table A1:** Codebook used for the data analysis: list of codes and their corresponding sub-codes.

Codes	Sub-Codes
Actors	Associations and NGOs • Animal welfare associations and NGOs • Cellular agriculture associations and NGOs • Consumer associations and NGO • Dairy processor associations • Environmental associations and NGOs • Farmers’ associations • Fish and seafood processor associations • Meat processor associations • Others Chain actors—Distribution • Cafes • Distributor • Hotel • Online retailers • Physical retailer • Restaurant • Retailers • Trader Chain actors—Production • Dimension • SMEs • Start-ups • Large enterprises • Sector • Feed • Meat • Seafood • Type of actor • CM processors • Suppliers of raw material • Suppliers of technology Institutional actors • EU • International Institution • National Governments • National Agencies • Agriculture fisheries • Environment and animal welfare • Food safety • Regulatory bodies • Research and innovation • Trade and export Other actors • Conventional Meat Processors • Farmers • Private Research Public Research
Culture	Actor involved in creating perceptions • Influencers • Media Creating expectation Social norms • Regarding citizens—societal acceptance • Regarding R&D • Regarding production and selling Success stories • International reputation • Visible success of CM companies Wealth generation
Finance	Barriers to funding Beneficiaries of funding Funding from NGOs and associations Private funding • Angel investors • Bankers • Celebrity investors • Big agrifood companies • Venture capitals • Private equity companies • Investment companies Public funding • EU • International • National • Regional Trends in funding Type of financial products • Grants • Investments • Loans • Micro-loans • Series A • Series B • Seed Funding Type of relationship between financial actors and beneficiaries • High complexity of transaction • High information asymmetry • High monitoring and control • Low complexity of transaction • Low information asymmetry Low monitoring and control
Geographical Location	Africa Asia • Asia-Pacific • China • India • Israel • Japan • Singapore • Thailand Cross continent Europe • Belgium • Cross country • Denmark • European Union • Finland • France • Germany • Ireland • Italy • Poland • Portugal • Spain • The Netherlands • United Kingdom North America • Canada • USA Oceania • Australia • New Zealand South America Brazil
Human Capital	Degrees of employees Professional roles Role of educational institutions Skills and capabilities
Market	Business associations Characteristics of the market Competitiveness in the market Market potential Merge and Acquisition Price and affordability • Consumers’ acceptance • Price to consumers Production cost • Energy • Raw materials • Skills • Storage • Technology and infrastructure Production model Value chain • Actors’ role in the value chain • Governance of the value chain • High-income vs. low-middle income countries Structure of the value chain
Policy	Governmental support Regulations • Food safety • Gaps in regulation • GMO • Information and data provision • Labelling • Marketing (selling and consumption) • Meat ontology • Patent and trade secret • Production • Promotion • Research Politics—Lobbies • Against • Favourable
Support	Accelerators and incubators Access to technological innovation • Closed—Patent • Open Consultancy • CM processors • Private research • Public research Energy • Amount of energy used • Source of energy Events • Conferences • Cooking—Taste event • Public campaigns • Public meetings Other infrastructures • Telecommunications • Transportation and logistics Technology and R&D • Actors involved in technology development • State of technology • Technology companies

## Appendix B

### Appendix B.1. Temporal Distribution of the Studies

The literature review proves cultured meat to be a recent topic in agrifood economics and policies, as all studies were published after 2013. As many authors pointed out, 2013 saw a turning point for cellular agriculture as the first cultured hamburger was presented to the public [56,58,74]. Publications were limited to a small number until 2020. The period 2021–23 saw a peak in the number of publications on the topic, with a stable trend of between 10 and 11 publications per year (Figure A1).

### Appendix B.2. Type of Publishing Journal

The articles are well distributed among different journals, covering topics such as food systems, nutrition, technology, and law. Only six journals published more than one article, with *Frontiers in Sustainable Food System* publishing four articles (Table A2).

**Table A2:** Number of studies published per journal. Book chapters are excluded.

Journal	No. of Studies
*Frontiers In Sustainable Food Systems*	4
*Agriculture And Human Values*	3
*Technology In Society*	2
*Frontiers In Nutrition*	2
*Journal Of Rural Studies*	2
*Future Foods*	2
*Nanoethics*	1
*Journal Of Cultural Economy*	1
*Food And Drug Law Journal*	1
*Humanities & Social Sciences Communications*	1
*Food Control*	1
*Journal Of Cleaner Production*	1
*Food Culture & Society*	1
*Law, Innovation And Technology*	1
*Food Policy*	1
*Technological Forecasting And Social Change*	1
*Food Research International*	1
*International Food And Agribusiness Management Review*	1
*Foods*	1
*Journal Of Animal Science And Technology*	1
*Annals Of Animal Science*	1
*Journal Of Consumer Behaviour*	1
*Biosocieties*	1
*European Food And Feed Law Review*	1
*Canadian Journal Of Women And The Law*	1
*Meat Science*	1
*Research Policy*	1
*Geoforum*	1
*European Journal Of Risk Regulation*	1
*Geographical Journal*	1
*Future Of Food: Journal On Food, Agriculture And Society*	1

### Appendix B.3. Methodological Approaches of Selected Studies

The 43 studies selected can be divided into two groups according to the methodological approach to data and information development. The first group (23 studies) includes research papers based on methodological-based data collection. This delineation implies a research rigour in the methodologies employed in data acquisition, administration, and analysis within these studies. This group of research studies adopted various methodological approaches, and both primary and secondary data were collected (Table A3). Overall, most of the reviewed studies adopt qualitative approaches, especially semi-structured interviews with stakeholders such as researchers, CM companies, policymakers, and NGO representatives. The authors proceeded by analyzing data using their content (four articles) and thematic analysis (five articles). The articles applying secondary data used sources such as patent databases, websites, videos, news, and transcripts from public meetings. Furthermore, there were 11 studies applying theoretical frameworks to frame their analysis. Two studies applied the Sociology of Expectations to analyze how expectations of the future influence the present situation of the CM market [56,57]. Other examples of theoretical frameworks applied include the following: the Strategic Niche Management and Multi-Level Perspective framework to analyze the development of the CM sector from the perspective of socio-technical transitions [35]; the Transformative Innovation Policy approach to investigate politicians’ perceptions of CM [36]; the GVC framework to analyze the capabilities required to enter the CM GVC [46]; and the combination of the GVC and EE to study the Singaporean Entrepreneurial Ecosystem for CM start-ups [43].

**Table A3:** Methodological approaches adopted by research papers.

Methodological Approach	No. of Studies
Qualitative study; Primary data	9
Qualitative study; Secondary data	6
Qualitative study; Primary and secondary data	3
Literature review	3
Quantitative study; Primary data	2

The second group (20 studies) included secondary literature pieces. This heterogeneous group includes various types of studies such as the following: (1) multidisciplinary informative reviews that present a broad overview of the topic, discussing multiple aspects based on notions from the past literature and also presenting new insights into existing problems and future perspectives; (2) perspective studies that offer unique viewpoints on the topic in question based on references from the literature and personal opinions; (3) legislative reviews to compare regulatory frameworks on the topic and the inclusion of opinions on the future development of legislation; and (4) opinion articles that discuss the merits and drawbacks of a hypothesis or scientific theory and rely on constructive criticism to present the authors’ position and challenge the current state of knowledge of the topic in discussion. These types of studies were distributed differently in our sample, with a vast majority of multidisciplinary informative reviews (Table A4). These studies are included in the present literature review because, first, they are articles offering specific viewpoints or fresh perspectives on existing research within a specific field; second, the CM chain is still in its infancy and going through a process of definition and thus may benefit from studies that provide critical elements to shape its interpretative framework and conceptual definition.

**Table A4:** Number of secondary studies included in the reviewed literature by type.

Type of Secondary Study	No. of Studies
Multidisciplinary informative review	9
Legislative review	5
Opinion article	4
Perspective	2

### Appendix B.4. Type of Animal Protein Addressed

While the current literature search aimed to cover all types of products reproducing animal protein, the reviewed articles focus exclusively on cultured meat, except for one study, which entirely dedicated its study to cultured dairy [75]. Nonetheless, while discussing CM companies, some studies mention companies which produce other cultured animal proteins, such as seafood and foie gras [40,41]. Thus, the authors of the present article decided to align with the reviewed literature and use the expression “cultured meat” in a general way to include references to companies producing cultured seafood and dairy.

## Appendix C

The studies published between 2021 and 2023 contain more than 75% of the occurrences (Figure A3). All the EE themes occurred in the reviewed literature since the first publications in 2013, except for market and human capital. While market capital occurred first in an article published in 2014, the domain of human capital has only been addressed after 2020, showing a remotely new interest in this topic.

Policy is the most geographically focused domain, demonstrating a strong co-occurrence with geographical location (Figure A4). This is accurate since the majority of the citations on policy focused on the regulations of individual countries.
